# Correlation-Based Network Analysis of Metabolite and Enzyme Profiles Reveals a Role of Citrate Biosynthesis in Modulating N and C Metabolism in *Zea mays*

**DOI:** 10.3389/fpls.2016.01022

**Published:** 2016-07-12

**Authors:** David Toubiana, Wentao Xue, Nengyi Zhang, Karl Kremling, Amit Gur, Shai Pilosof, Yves Gibon, Mark Stitt, Edward S. Buckler, Alisdair R. Fernie, Aaron Fait

**Affiliations:** ^1^Institute of Dryland Biotechnology and Agriculture, Jacob Blaustein Institutes for Desert Research, Ben-Gurion University of the NegevMidreshet Ben-Gurion, Israel; ^2^Institute for Genomic Diversity, Cornell UniversityIthaca, NY, USA; ^3^Mitrani Department of Desert Ecology, Blaustein Institutes for Desert Research, Ben-Gurion University of the NegevMidreshet Ben-Gurion, Israel; ^4^Max Planck Institute of Molecular Plant PhysiologyGolm, Germany

**Keywords:** *Zea mays*, correlation-based network analysis, metabolic networks and pathways, enzymatic processes, metabolism, TCA cycle

## Abstract

To investigate the natural variability of leaf metabolism and enzymatic activity in a maize inbred population, statistical and network analyses were employed on metabolite and enzyme profiles. The test of coefficient of variation showed that sugars and amino acids displayed opposite trends in their variance within the population, consistently with their related enzymes. The overall higher CV values for metabolites as compared to the tested enzymes are indicative for their greater phenotypic plasticity. H^2^ tests revealed galactinol (1) and asparagine (0.91) as the highest scorers among metabolites and nitrate reductase (0.73), NAD-glutamate dehydrogenase (0.52), and phosphoglucomutase (0.51) among enzymes. The overall low H^2^ scores for metabolites and enzymes are suggestive for a great environmental impact or gene-environment interaction. Correlation-based network generation followed by community detection analysis, partitioned the network into three main communities and one dyad, (i) reflecting the different levels of phenotypic plasticity of the two molecular classes as observed for the CV values and (ii) highlighting the concerted changes between classes of chemically related metabolites. Community 1 is composed mainly of enzymes and specialized metabolites, community 2′ is enriched in N-containing compounds and phosphorylated-intermediates. The third community contains mainly organic acids and sugars. Cross-community linkages are supported by aspartate, by the photorespiration amino acids glycine and serine, by the metabolically related GABA and putrescine, and by citrate. The latter displayed the strongest node-betweenness value (185.25) of all nodes highlighting its fundamental structural role in the connectivity of the network by linking between different communities and to the also strongly connected enzyme aldolase.

## Introduction

Metabolic networks are represented in databases of genome-scale networks as relatively defined pathways (Tohge and Fernie, 2009; Fiehn et al., 2011), e.g., PlantCyc (http://www.plantcyc.org/) (Zhang et al., 2010a), BioCyc (http://biocyc.org/) (Karp et al., 2005), KEGG (http://www.genome.jp/kegg/) (Kanehisa et al., 2014), and MetRxn (http://ec2-54-213-167-41.us-west-2.compute.amazonaws.com/) (Kumar et al., 2012). Nevertheless, the schematically represented boundaries between series of biochemical reactions neglect the occurrence of crosstalk and coordinated regulation between biochemically distant pathways. Thus, considering metabolic pathways as stand-alone entities can be misleading in that they fail to grasp the full complexity of metabolic networks. Moreover, the representation of biochemical reactions as genome-scale networks requires *a priori* knowledge of their stoichiometric balance. Correlation-based network analysis (CNA), on the other hand, provides a method to illustrate the relationship between molecular components without prior knowledge of the underlying chemistry. The relational ties established between different cellular components via CNA can represent coordinated changes of abundances in response to a given genetic or environmental perturbation (Toubiana et al., 2013). Furthermore, the topology of correlation networks can be analyzed with well-defined network properties from graph theory and communities can be identified with community detecting algorithms (Newman and Girvan, 2004).

In the last few decades the natural variance of various species was exploited resulting in the generation of populations dedicated to the study of complex traits. For example a population of introgression lines from the cross of *L. pennelli* and *L. esculentum* cv. M82 (Eshed and Zamir, 1995) has proven to be an excellent tool for research in countless studies (Lippman et al., 2007) leading to the identification of quantitative trait loci and to the cloning of genes of agronomic and biological importance (see for example Frary et al., 2000; Fridman et al., 2004; Lippman et al., 2007).

In recent years there have been growing efforts to exploit association panels, which are designed to capture a wide phenotypic variability (Yu and Buckler, 2006; Scossa et al., 2016). This approach successfully elucidated the genetic basis of metabolic natural variance, including that of carotenoids, glucosinolate and organic acids in different plant species (Riedelsheimer et al., 2012; Gonzalez-Jorge et al., 2013; Lipka et al., 2013; Verslues et al., 2014).

Using the entire maize nested associated mapping (NAM) population (Buckler et al., 2009), composed of approximately 5000 recombinant inbred lines (RIL), Zhang et al. (2015) managed to map 12 essential C and N metabolites in the maize leaf. In another genome wide association study (GWAS), Zhang et al. (2010b) used a preliminary panel of eight diverse maize inbred lines to map nine different enzymes in the leaf. They then continued to use a subset of 101 lines, capturing the maximal genetic diversity, of the core of the 300-line association panel, resulting from the cross between the inbreds B73 × Mo17 (IBM) (Flint-Garcia et al., 2005), to further study NAD-dependent isocitrate dehydrogenase variations—the results suggested a single putative SNP. The same core population was also used for the current study to investigate the phenotypic variation of 43 metabolites and 13 enzymes and their relationship to each other in the maize leaf. The different cellular compounds were chosen in association with C and N central metabolism. Also here, initially, a GWAS was carried out. However, often, the limited sample size, population structure, and/or cryptic relatedness of many plant populations targeted for GWAS can lead to weak associations between a trait and its prospective locus (Astle and Balding, 2009). Thus, rendering no viable results. Alternatively, a CNA may be applied to describe the relationship between different cellular structures as has been done in a plant biomass study in *Arabidopsis* (Sulpice et al., 2010). Similarly, in the present study, a CNA approach was applied to investigate the phenotypic variation of metabolite and enzyme levels. The integration of these results provides insights into the putative relationships in leaf metabolism and specifically highlights the central role of citrate in the maintenance of C-N metabolism.

## Materials and methods

### Population and greenhouse experiment

For the analyses conducted here a subset of the Flint-Garcia et al. (2005) intermated recombinant inbred lines (IRILS) from the intermated B73 × Mo17 (IBM) cross was used—in total 101 lines. Using five replicates on average per line, plants were grown in cell-packs in the green-house in a completely randomized design. For each line three seeds were sown in each cell and thinned 5 days after germination to one plant per cell, ensuring uniform germination across the experiment. At 35 days after germination, tissue was collected from the youngest expanded leaf and immediately frozen in liquid nitrogen. The collected tissue was stored at −80⋅C until analysis.

### Metabolite profiling and enzyme assays

Relative metabolite content (Supplementary data 1) was determined by gas chromatography-mass spectrometry essentially as described in Roessner et al. (2001) and Lisec et al. (2006). Enzymatic assays (Supplementary data 2) were performed as described in Zhang et al. (2010b) and Zhang et al. (2010c). Metabolites and enzymes were chosen based on their involvement in central pathways of C and N metabolism.

### Data processing and statistics

Metabolite data generated by GC-MS are composed of unique mass intensity values for each annotated compound. The raw data for each metabolite was normalized by dividing each value by its corresponding control compound ribitol, recorded for each chromatogram. Raw enzyme activity was standardized by plate mean followed by normalization for each enzyme as the difference between the standardized enzyme activity and the overall mean-activity. The resulting dataset, composed of 5.7% missing data, was completed by data imputation (Stacklies et al., 2007). For the estimation of random and fixed effects, best linear unbiased prediction (BLUP—Supplementary data 3) values were calculated for the metabolite and enzyme profiles and used in all subsequent analyses. In addition, to test for variable dependencies and shared variance, all variables were correlated using the Pearson product-moment correlation (Supplementary data 4), describing the linear dependency of two variables. The shared variance is estimated by squaring the resulting Pearson correlation coefficient. All statistics were calculated with R statistical software (R Development Core Team, 2009).

### Coefficient of variation and broad-sense heritability (H^2^) test

The coefficient of variation is defined as the ratio of the standard deviation to the mean and was calculated accordingly. H^2^ is defined as the proportion of the genetic variation from the total phenotypic variation and was calculated as described in Zhang et al. (2010b). H^2^ analysis results were arranged into bins of 0.1 intervals.

### Network analysis

The generation of the network was based on the correlation analysis of all metabolites and enzymes. All components were tested for normal distribution across all parental and inbred lines by employing a Shapiro-Wilk test. Invariably, the assumption of normal distribution was violated for all metabolites. Thus, the non-parametric Spearman rank correlation was chosen to produce correlation coefficients.

To construct the network, first the *p*-value threshold (≤ 0.011) corresponding to a *q*-value of 0.05 was determined. Second, the adequate correlation coefficient threshold was chosen by testing the stability of four different network properties, i.e., average node degree, clustering coefficient, network density, and diameter across a range of *p*-values. For a full description on these network properties the reader is referred to Toubiana et al. (2013). The correlation coefficient, at which the network displayed a robust behavior in all four properties, across a range of *p*-values, was chosen as the threshold for network construction. In other words, the correlation coefficient was set once the values of the network properties did not change across different *p*-values ranging from 0.01 to 0.05. For the current research a correlation coefficient of ≥0.3 was estimated as the appropriate threshold.

Subsequently, the network clustering into communities was achieved by employing the walktrap community detecting algorithm (Pons and Latapy, 2005). The communities were detected based on a non-weighted version of the graph, not integrating the correlation coefficient for the links. The statistical significance of the communities with more than four nodes was tested by performing a Wilcoxon signed rank test. The test was performed by assessing the degree of node-connectivity (Toubiana et al., 2013) of the isolated community as compared to the degree of the nodes of the community still embedded in the network following the subtraction of the community specific edges. The size of a node in the network reflects its degree of connectivity. The relative width and color of a link represent the absolute size of the correlation coefficient and its sign, respectively (blue = positive correlation coefficient, red = negative correlation coefficient). For the analysis of nodes, we estimated the node connectivity (nodal degree) and node betweenness properties (Freeman, 1977), the latter property of a node *i* is given by the number of geodesic distances between two nodes that contain that node. The geodesic distance between two nodes *i* and *j* is the length of a shortest path between them. Significance of the estimated values was determined by permutation tests of the correlation network with 10,000 iterations.

All computations for network visualizations were generated in R (R Development Core Team, 2009) The software Cytoscape (Shannon et al., 2003) version 2.8.3 was used for network visualization. Network properties and communities were computed by the igraph R package.

## Results

**Figure 1:**
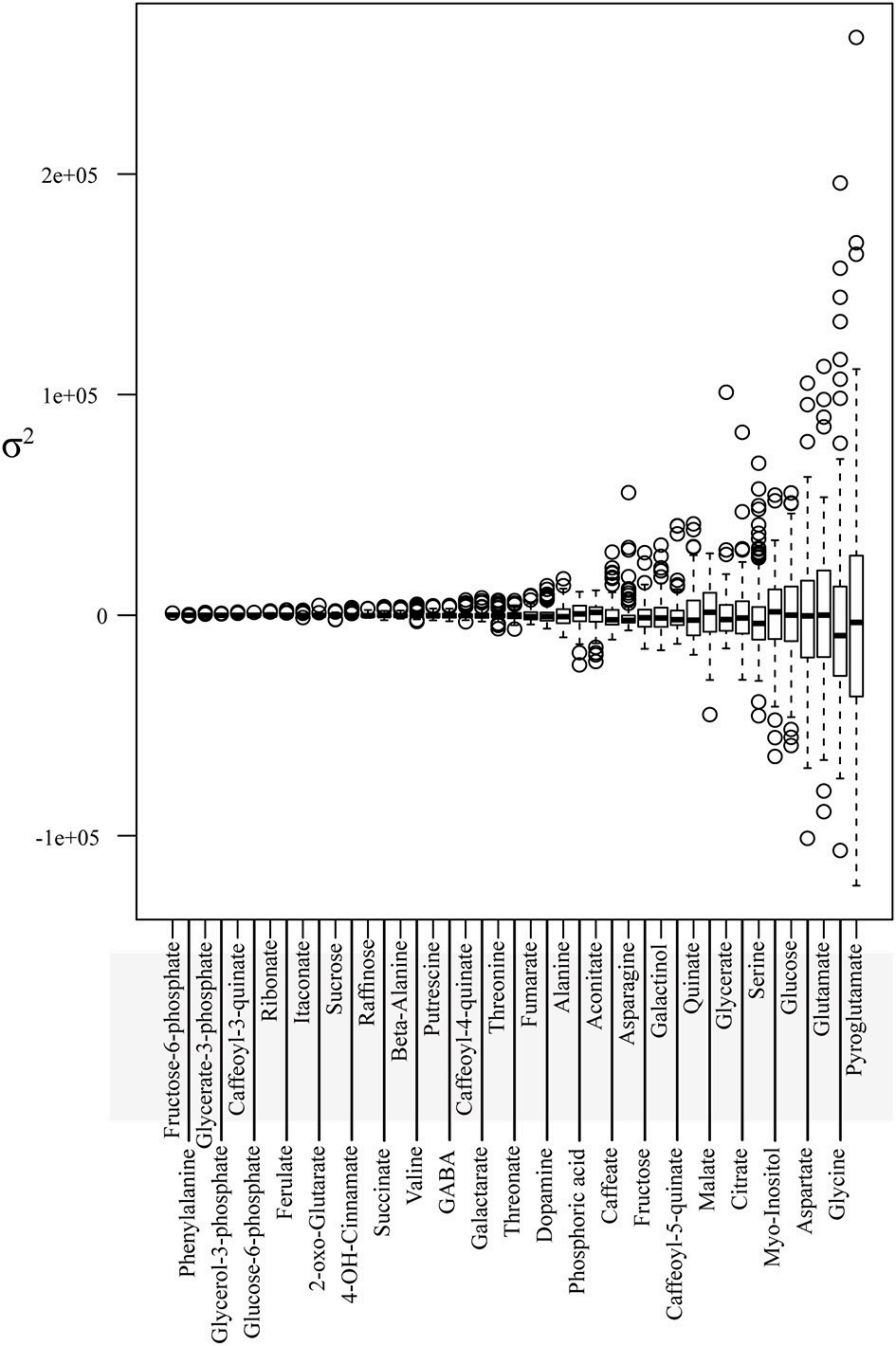
**Metabolite profiles descriptive statistics**. Boxplot of metabolite BLUP value profiles of the core subset of the IBM population. Metabolites are sorted in ascending order along the x-axis according to the estimated variance.

**Figure 2:**
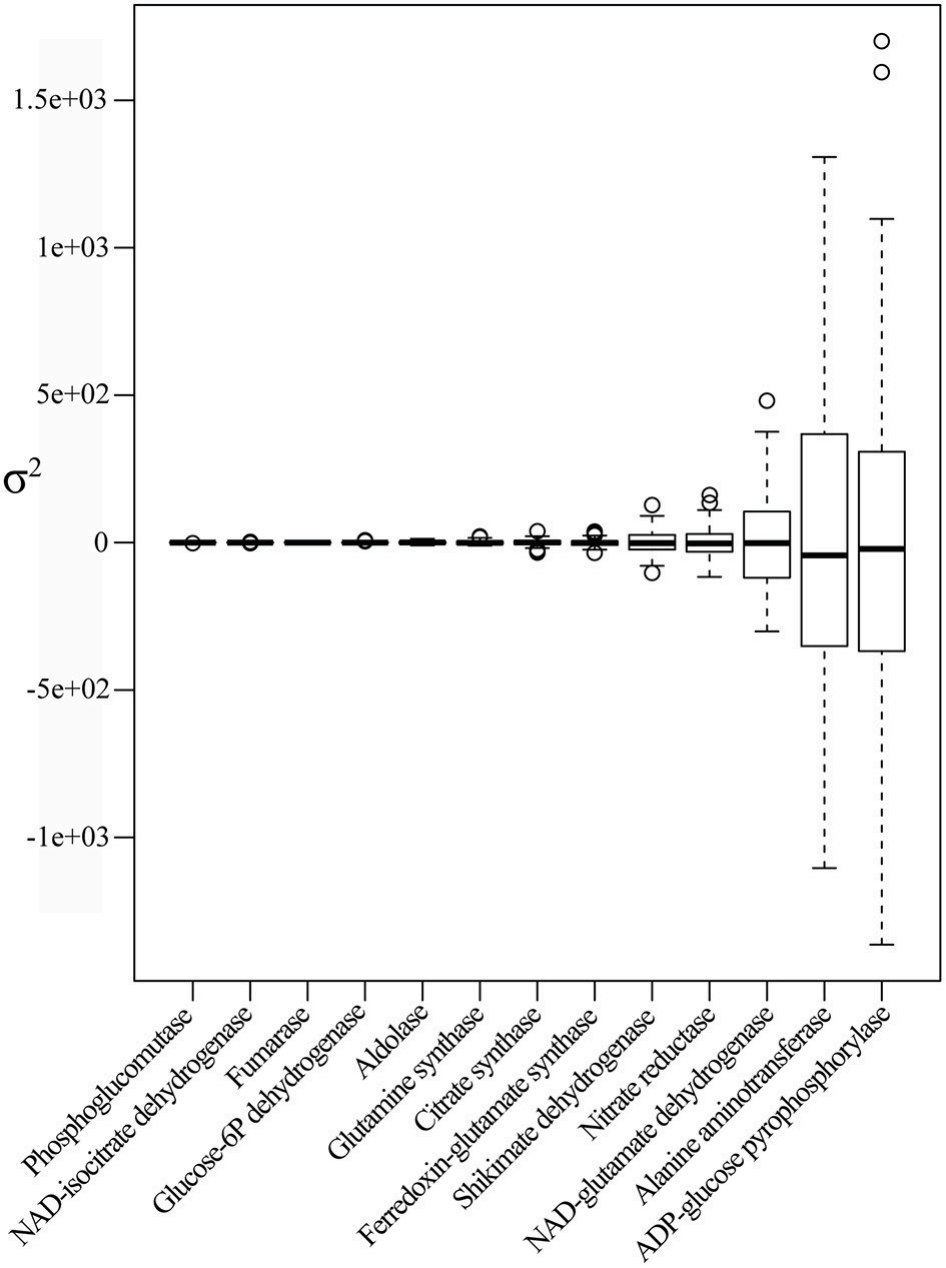
**Enzyme profiles descriptive statistics**. Boxplot of enzyme BLUP value profiles of the core subset of the IBM population. Enzymes are sorted in ascending order along the x-axis according to the estimated variance.

The understanding of C-N metabolism and its underlying genetic regulation of C4 plants is a key aspect for the amelioration of crop plants toward higher yields (Zhang et al., 2015). In the current study we made use of the maize IBM subset collection to measure the relative content of metabolites and enzymes associated with C-N metabolism in the leaf. In total, we unequivocally identified and measured 43 metabolites of central metabolism and 13 enzymes related to it. First, we standardized and normalized all enzymes and metabolites (for details see Materials and Methods: data processing and statistics) and then calculated the corresponding averages and variances, illustrated as boxplots (Figures 1, 2, respectively). Metabolite variance ranged from ~9.450e+03 to ~3.746e+09. On the lower extreme of the variance spectra were fructose-6-phosphate, glucose-6-phosphate, glycerate-3-phosphate, and glycerol-3-phosphate (Figure 1). By contrast, amino acids, including pyroglutamate, glycine, glutamate, and aspartate, showed the highest variance within the population.

The enzyme variance ranged from 0.34 to ~3.381e+05. Phosphoglucomutase, an essential enzyme in glycogenesis, exhibited the lowest variance, while ADP-glucose-phosphorylase, an essential enzyme in starch synthesis, revealed the greatest variance, followed by alanine aminotransferase and NAD-glutamate dehydrogenase, two enzymes involved in N-metabolism (Figure 2).

### The IBM population shows significant phenotypic plasticity in central N assimilation processes

Next, we estimated the coefficient of variation (CV) of the two datasets (Supplementary data 5). The CV is defined as the standard deviation over the mean and by that reveals the variability of a variable in relation to its mean. For biological data it allows insights into the phenotypic plasticity (Elowitz et al., 2002); in other words, the greater the CV the greater the phenotypic plasticity. Metabolites showed overall higher variance (variability) than enzymes. However, when estimating the CV in the joint dataset, eight of the 13 enzymes displayed the greatest CV values, suggesting for a significantly greater plasticity of these enzymes across the population as compared to metabolites. These enzymes are: shikimate dehydrogenase, citrate synthase as well as glutamine synthase, and ferredoxin-glutamate synthase, alanine aminotransferase NAD-glutamate dehydrogenase, nitrate reductase and ADP-glucose pyrophosphorylase (Supplementary data 5). Whilst it cannot be speculated if the variation in enzyme activity lies in the genetic diversity or in the interaction between the genetics and the environment, the evident occurrence in the list of major enzymes involved in N-assimilation together with citrate synthase suggests the importance of a significant tuning of the N-C metabolism.

**Figure 3:**
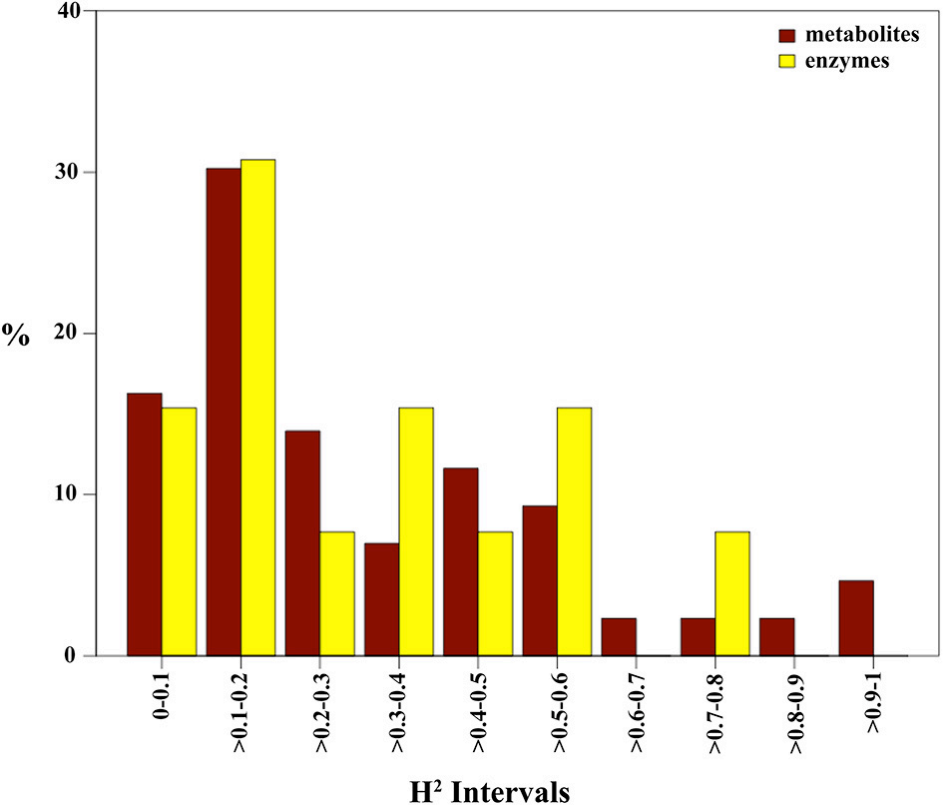
**Broad-sense heritability of maize metabolites**. Broad-sense heritability (H^2^) values were calculated for all metabolites and enzymes of maize leaves in the background of the IBM population. Values of H^2^ were divided into bins of 0.1 intervals. Bars represent the relative number for each respective bin.

To explore the level of heritability of metabolites and enzymes, broad-sense heritability values (H^2^) were computed. H^2^ estimates were divided into 10 bins of 0.1 intervals for which the relative frequencies are given (Figure 3). H^2^ values approaching one suggest for an increasingly unperturbed link between genotype and phenotype. The H^2^ bar-plot reveals a positive skewness for both metabolites and enzymes suggestive for a consistent environmental impact or genetic-environment interaction on their level. Indeed, 34 of the 43 (~79%) identified metabolites and 10 of the 13 enzymes (~77%) exhibit an H^2^ score below 0.5 (Supplementary Tables 1,2). Only nine metabolites displayed an H^2^ score greater than 0.5. Interestingly, galactinol and asparagine were the highest scorers among metabolites, with values of 1 and 0.91, respectively (Supplementary Table 1). For the enzymes, only nitrate reductase (0.73), NAD-glutamate dehydrogenase (0.52), and phosphoglucomutase (0.51) revealed H^2^ values above 0.5.

### Network analysis reveals a tightly interconnected amino acid community and its relationships to carbon metabolism

Genome-scale metabolic networks are presented as hypergraphs (Klamt et al., 2009; Larhlimi et al., 2011), where one link may connect several nodes based on stoichiometric calculations. The relationships between cellular components may also represent coordinated behaviors, which are captured in correlation-based networks. As such, here, we employed a correlation-based network approach based on the integrated dataset, i.e., metabolite-to-metabolite, metabolite-to-enzyme and enzyme-to-enzyme associations are visualized by a graph of nodes and links. Links represent the correlation coefficient between any two nodes. For a correlation coefficient to be integrated into the network, threshold tests need to be passed (see Materials and Methods). For the current network, 40 metabolites and 10 enzymes (nodes) showed 135 (links) significant relations to each other. Variables corresponding to any two adjacent nodes were tested for variable dependencies and shared variance by testing for the linear relationships employing the Pearson product-moment correlation. Out of 135 connections, 83 showed an absolute linear dependency of ≤ 0.4, 43 a linear relationship between 0.4 and 0.6, and 9 greater than 0.6 (Supplementary data 3). 100 connections revealed a shared variance of ≤ 20%, 29 a shared variance between 20 and 40%, and 6 greater than 40% (Supplementary data 3).

The nodes in the network where then grouped into communities applying the walktrap community algorithm (Pons and Latapy, 2005), identifying densely connected subgraphs (i.e., communities) based on short random walks. The community detection analysis revealed the presence of five communities, of which communities 1, 2, and 5 (Supplementary Figure 1) showed salient structures as computed by community significance estimations (see Materials and Methods), resulting in *p*-values of community 1 < 0.001, community 2 < 0.0001, community 3 = 0.065, community 4 = 0.26, and community 5 = 0.015. Because communities 3 and 4 showed to exhibit non-significant structures but at the same time maintain a number of bonds to communities 2 and 5, we combined community 2 with community 4 (community 2′ in Figure 2), creating a community of mainly N-compounds and phosphates, and community 3 with community 5 (community 3′ in Figure 2), creating a community of organic acids and sugars. After repeating the community significance test, we obtained *p*-values for community 2′ < 0.00001 and for community 3′ < 0.0001, respectively, indicative for their structural significance.

Community 1 contains 9 of the 10 enzymes present in the network and three of the specialized metabolites. All nodes within the community are tightly interconnected, suggestive for a coordinated behavior for most of the analyzed enzymes. Except for aldolase all edges incident on the remaining nodes are of positive correlations. Aldolase connects via 11 edges of negative correlations to adjacent nodes, of which six connect to other enzymes of the same community and the remaining five edges connect to metabolites. Community 2′ is composed entirely of 19 metabolites, interconnected via 50 edges. It is particularly enriched in N-compounds with ten of the twelve present amino acids in the network and putrescine, which shows a significant positive correlation of 0.44 to its product, the non-proteonogenic amino acid γ–aminobutyric-acid (GABA). The amino acids are tightly connected to each other, indicative for the highly branched and interconnected pathways of amino acid biosynthesis (Less and Galili, 2009). The homogeneous sizes of the amino acid nodes unveil a balanced distribution of connections between them. Furthermore, community 2′ embeds all phosphates present in the network, representative for the pentose phosphate pathway (PPP) and the Calvin cycle. Several amino acids in community 2′, specifically glutamate, connect to nodes in community 3′, which incorporates two sugars and six organic acids. The sound positive correlation (*r*=0.82) between the two sugars, glucose and fructose, emphasizes their close biochemical relatedness. The bonds between the amino acids in community 2′ and certain organic acids and sugars in community 3′ suggest for the close biochemical relationship and interdependence of the amino acid metabolism, the tricarboxylic acid (TCA) cycle, and glycolysis. For example, the enzyme nitrate reductase, aspartate, citrate and fumarate, the latter connected to malate all clustered to community 3′. Moreover, the coordinated pattern of change NAD-isocitrate dehydrogenase (NAD-IDH) in community 1 with the metabolites GABA and putrescine of community 2′ on the one hand and with citrate synthase and fumarase on the other, reflect the highly coordinated balance between N assimilation and C metabolism. Within this context the absence of direct links between metabolites of the TCA cycle and the cycle enzymes is notable. That said the results collectively reflect a highly coordinated N assimilation and C metabolism. Furthermore, the network highlights the strong negative relationship of the enzymes aldolase and phosphoglucomutase, two main players in the glycolysis, in community 1 and their consequent inverse relation with citrate in community 3′.

### Network property analysis suggest for a central role of citrate in the leaf metabolite-enzyme network validated by transcript analysis

To identify which nodes within the network could be of particular relevance in the structure of the network, we estimated the node connectivity and node betweenness properties for all nodes. Our findings show that aldolase, threonine, and citrate exert the highest node degree in the network—each with a connectivity of 11 (*p*-value < 0.001) as compared to an average node degree of 5.4. The node betweenness reflects the level of integration of a node, between communities, within the network (see Materials and Methods or Toubiana et al., 2013 for definition). Citrate with 185.25 (*p*-value < 0.001) displayed the highest node betweenness of all nodes in the network, followed by aldolase with 171.25. Threonine exhibited a node betweenness of 47.48. The average node betweenness of the network was estimated as 40.12. The outstanding node betweenness of citrate suggests a central role for this TCA cycle intermediate in the network. In other words, most of the shortest paths between any two nodes in the network pass through citrate. The central role of citrate and its connections to central N-compounds, such as glutamate and aspartate on the one hand and to enzyme aldolase (displaying second highest node betweenness) and organic acids on the other hand is demonstrative for its role as a modulator between C-N metabolism.

## Discussion

The profiling of metabolites and enzyme activities across a maize association panel population was implemented and subjected to network analysis. An attempt to identify loci for the molecular traits measured using a genome wide association mapping was however unsuccessful. Central metabolism is characterized by a complex multilevel regulation, redundant pathways in multiple cellular compartments, futile cycles and enzyme promiscuity (Collakova et al., 2012) all which contribute to its metabolic stability (Rontein et al., 2002), and which are likely the reason for the relatively low measured trait heritabilities (Figure 3). It is therefore not surprising that this and other studies were not successful to identify a significant link between the features analyzed and their underlying genetics. Having said that, recent studies in an introgression line population revealed the strength of CNA to aid in discerning the regulation of central metabolism (Toubiana et al., 2015).

**Figure 4:**
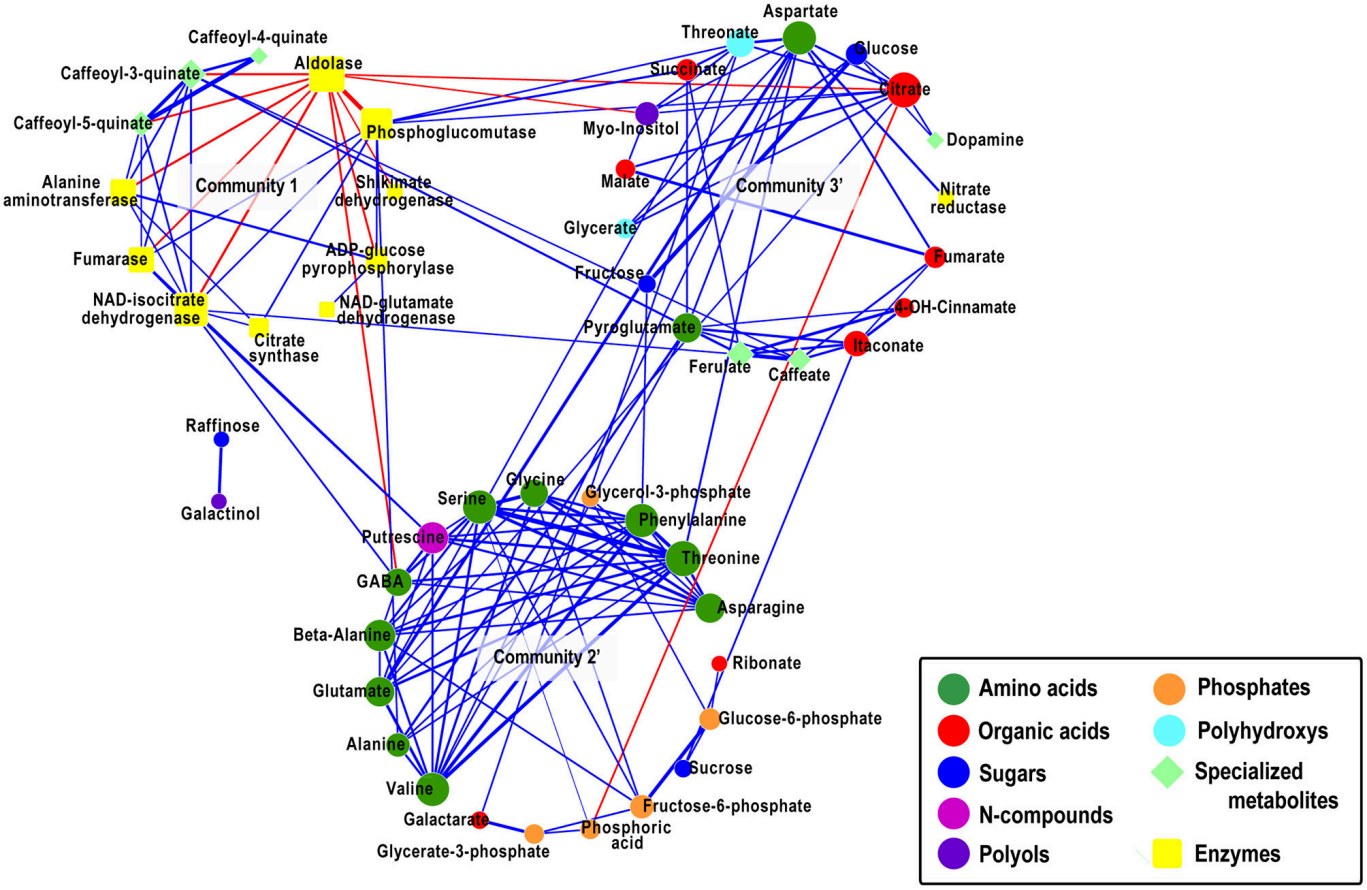
**Correlation-based metabolite network with combined communities**. Network visualization of metabolites as analyzed on the IBM population. Metabolites are presented as nodes and their relations as links. Computations of the correlations were conducted under the R environment. Cytoscape was used to generate graphical output of network. The Spearman rank correlation was employed to compute all pairwise correlations between metabolites across the entire set of inbred lines. Solely significant correlations are depicted (q ≤ 0.05 an *r*-value of ≥ 0.3). The relative width of edges corresponds to the absolute value of the estimated correlation coefficient. Positive correlations are denoted as blue edges, negative correlations are denoted as red edges. To distinguish between metabolites and enzymes in the network, nodes are represented in different shapes (metabolites = elliptical, enzymes = rectangular). Furthermore, nodes representing metabolites are color-coded according to their compound classes. The size of nodes corresponds to their relative connectedness (node degree). Metabolites are color-coded and clustered according to the walktrap community algorithm. The statistical significance of the occurrence of a community with more than four nodes was tested by performing a Wilcoxon signed rank test. The test was performed by assessing the degree of node-connectivity of the isolated community as compared to the nodes of the community still embedded in the network of which all community specific edges have been subtracted. Non-significant communities were combined with significant communities.

Here, we explore the relational ties between enzymes and metabolites implementing CNA to construct a unipartite network (Figure 4). Although the detected absolute correlation coefficients of the relationships between metabolites and enzymes ranged between values of 0.3 to 0.4—indicating rather moderate correlations—further investigations allowed us to suggest structures resembling known metabolic pathways.

Arranging the enzymes and metabolites into communities, as suggested by a community detecting algorithm, followed by statistical analysis, three salient, statistically sound communities were detected. All enzymes found in the network are embedded in community 1 revealing mainly positive correlations to each other indicative for a C-N metabolism co-regulation as also observed in tobacco plants (Scheible et al., 2000) and maize seedlings (Zhang et al., 2010c). Community 2′ is enriched in amino acids. Amino acids are regulated to a great extent (Galili and Hofgen, 2002) and their tight co-regulation was observed in different species including *Arabidopsis* (Sulpice et al., 2010) and tomato seeds (Toubiana et al., 2015). Community 3′ contains a number of compounds classes, amongst others TCA cycle intermediates and only one proteinogenic amino acid, aspartate. The interweavement of TCA cycle intermediates with compounds from other compound classes is one of its salient features being actively involved in a wide array of pathways of the metabolic network (Sweetlove et al., 2010) supplying carbon skeleton to amino acid biosynthesis, fatty acid metabolism, integrating N metabolism in the form of glutamate and GABA through 2-oxoglutarate and succinate, respectively (Fait et al., 2008), and the obvious intimate link to the glycolysis. The multiple connections between the three communities is exemplified by the centrality of the TCA cycle intermediate citrate in the network as shown by (i) its outstanding network properties of high connectivity (11, *p*-value < 0.001) and node betweenness centrality (185.25, *p*-value < 0.001) as compared to the average network connectivity (5.4) and average network node betweenness (40.12) and (ii) its link to aldolase the second highest node in respect to connectivity and betweenness centrality. These results illustrate the intertwined crosstalk between the TCA cycle, PPP and glycolysis for the maintenance of carbon metabolism. The close inter-regulation shown for these pathways is guaranteed by the very physical association of the entire glycolytic pathway with the cytosolic face of the outer mitochondrial membrane (Giege et al., 2003). Recent studies show that citrate is not synthesized from newly fixed carbon, but is instead accumulated at night, and degraded in the light, to act as an immediate source of carbon for the synthesis of 2-oxo-glutarate and for NH_3_ assimilation in the GOGAT pathway and linking carbon metabolism, nitrogen assimilation and photorespiration (Scheible et al., 2000). Our results, showing the link between citrate, glutamate and aspartate, and the latter with nitrate reductase, further emphasize the biological relevance of their coordinated pattern of change. Related coordinated patterns were observed for transcripts coding for central metabolic enzymes such as nitrate reductase and citrate synthase in the leaves of tobacco transformants with decreased expression of nitrate reductase and in nitrate-deficient wild-type tobacco (Scheible et al., 2000). There, an inverse diurnal pattern was identified. Nitrate and nitrite reductase and phosphoenolpyruvate carboxylase were highest at the end of the night and decreased steeply during the day. Inversely, the levels of cytosolic pyruvate kinase, mitochondrial citrate synthase and NADP-isocitrate dehydrogenase were highest at the end of the light period. During the day citrate decreased in level, malate accumulated, while 30% of the assimilated nitrate was accumulated in glutamine, ammonium, glycine and serine. During the night these patterns were reversed.

**Figure 5:**
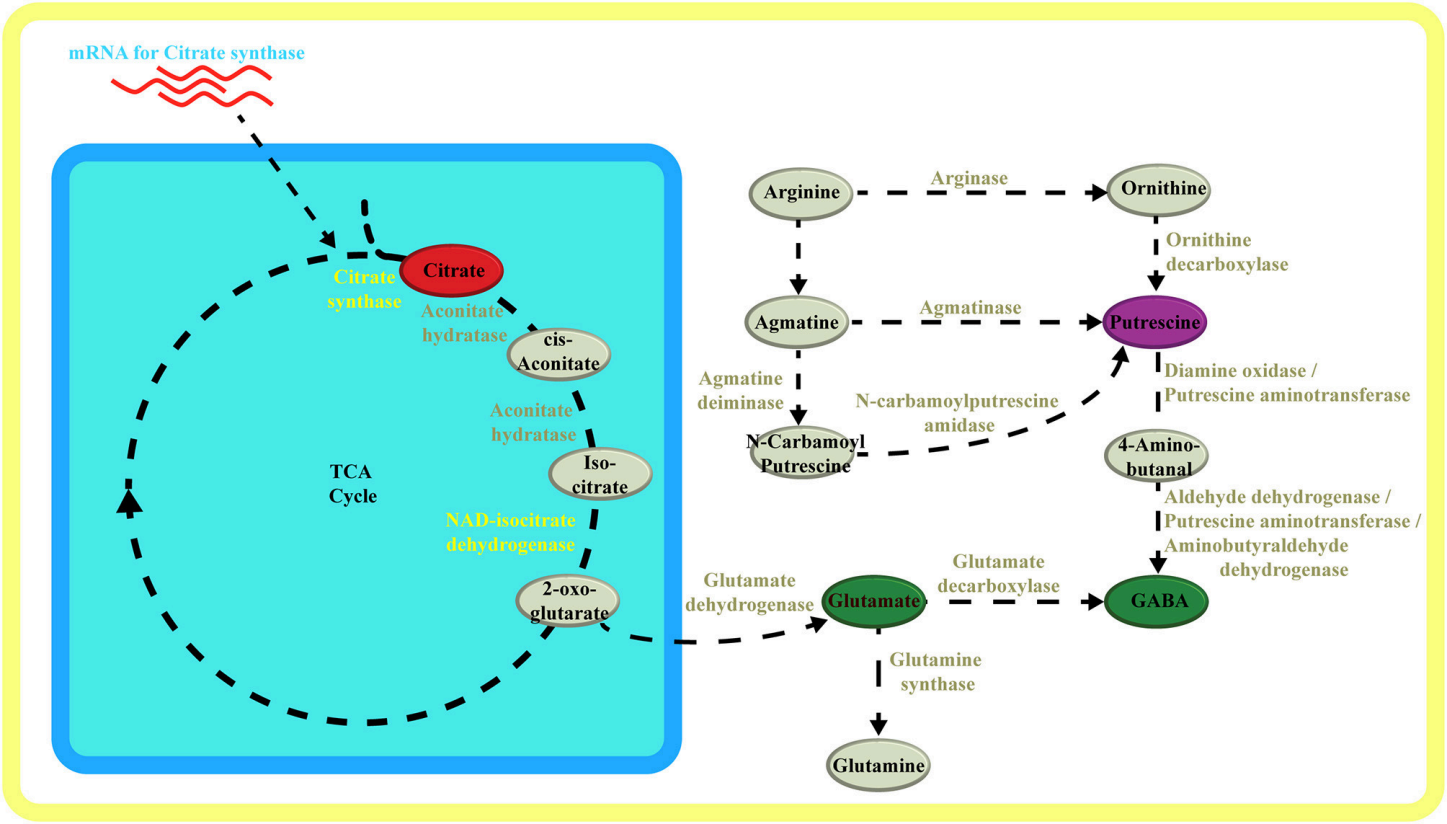
**Metabolic pathway schematic overview**. Schematic overview of metabolic pathways highlighting the TCA cycle in respect to glutamate, putrescine and GABA synthesis. Metabolites and enzymes are color-coded in accordance to compound classes and enzymes in the network (Figure 3, (Supplementary Figure 1). Adobe Illustrators was used to generate graphical output.

Last, the polyamine putrescine significantly correlated with the amino acid GABA. Both metabolites showed an interesting link with NAD isocitrate dehydrogenase, which could indicate a coordination of 2-oxo-glutarate biosynthesis with its integration within the GABA shunt both as a precursor of glutamate (via GDH) and as an amino acceptor (via GABA transaminase) (Figure 5). The pathway from putrescine to GABA is known (Ditomaso et al., 1992; Fait et al., 2008) and reference therein): putrescine can be converted via a two step oxidation into GABA (Figure 5). Though the pathway has been defined, only few other studies identified robust links between these two metabolites (Flores and Filner, 1985) and under stress (Shelp et al., 2012).

Our study exemplifies that the usage of CNA can lead to biologically sound conclusions on metabolic pathway structure and regulation and hypothesis generation given sufficient variance between data points, here represented by the natural variation characterizing the IBM maize population. Furthermore, the CNA based analysis of metabolite and enzyme profiles substantiates that citrate acts as a bridge between C and N metabolism.

## Author contributions

DT: statistical and network analysis and preparation of manuscript, WX: metabolic profiling, NZ: metabolic profiling, KK: GWAS analysis, AG: enzymatic assaying, SP: network analysis, YG: enzymatic assaying and manuscript preparation, MS: enzymatic assaying and manuscript preparation, EB: maize population and manuscript preparation, ARF: metabolic profiling and manuscript preparation, AF: metabolic profiling and manuscript preparation.

### Conflict of interest statement

The authors declare that the research was conducted in the absence of any commercial or financial relationships that could be construed as a potential conflict of interest.

## Supplementary material

Supplementary Figure 1 **Correlation-based metabolite network with communities**. Network visualization of metabolites as analyzed on the IBM population. Metabolites are presented as nodes and their relations as edges. The Spearman rank correlation was employed to compute all pairwise correlations between metabolites across the entire set of inbred lines. Solely significant correlations were chosen to be depicted. A permissive threshold level of *q* ≤ 0.05 an *r*-value of ≥ 0.3 was chosen for the identification of significant correlations. Metabolites are color-coded and clustered according to the walktrap community algorithm. The significance of the communities with more than four nodes was tested by performing a Wilcoxon signed rank test. The test was performed by assessing the degree of node-connectivity of the isolated community as compared to the nodes of the community still embedded in the network of which all community specific edges have been subtracted. Positive correlations are denoted as blue edges, negative correlations are denoted as red edges. The sizes of the nodes represent the relative degree of connectivity. Computations of the correlations were conducted under the R environment. Cytoscape was used to generate graphical output of network.

Supplementary data 1 **Metabolite raw profiles**.

Supplementary data 2 **Enzyme assay raw profiles**.

Supplementary data 3 **Metabolite and enzyme BLUP profiles**.

Supplementary data 4 **Correlation coefficients and ***r***^**2**^ values of Pearson correlation**.

Supplementary data 5 **Coefficient of variation of metabolites and enzymes**.

Supplementary Table 1 **Broad-sense heritability values of metabolites**.

Supplementary Table 2 **Broad-sense heritability values of enzymes**.
