# Supplementary figures and images for: Correlation-Based Network Analysis of Metabolite and Enzyme Profiles Reveals a Role of Citrate Biosynthesis in Modulating N and C Metabolism in Zea mays
Source: Front Plant Sci. 2016 Jul 12;7:1022. doi: 10.3389/fpls.2016.01022 (PMC4940414; doi:10.3389/fpls.2016.01022)

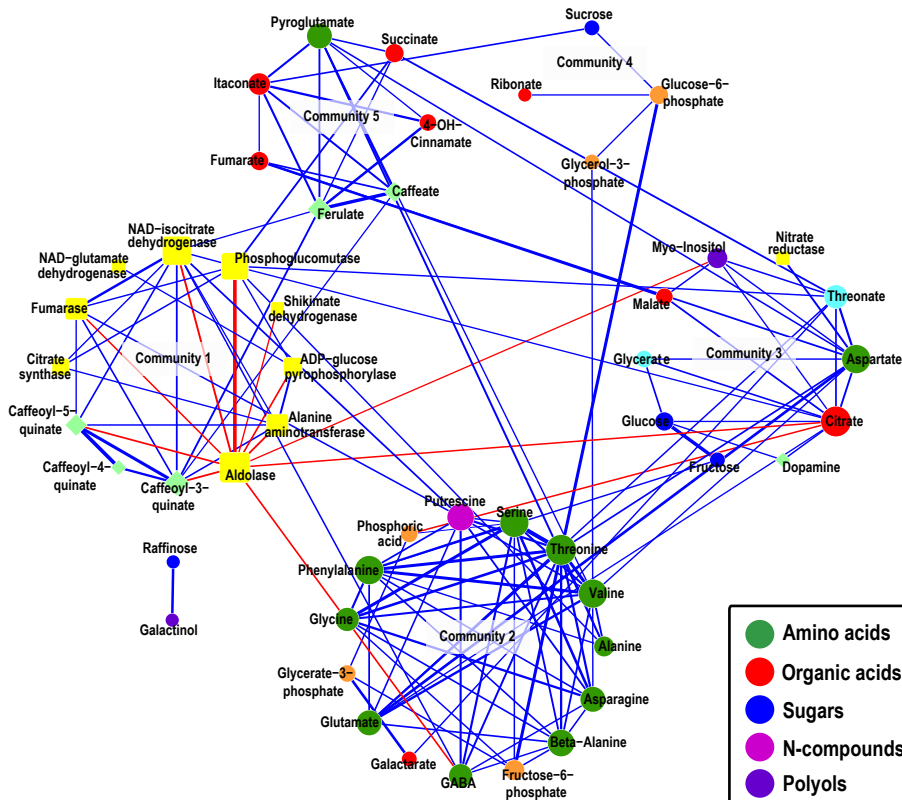

Supplement: Supplementary Figure 1 — Correlation-based metabolite network with communities. Network visualization of metabolites as analyzed on the IBM population. Metabolites are presented as nodes and their relations as edges. The Spearman rank correlation was employed to compute all pairwise correlations between metabolites across the entire set of inbred lines. Solely significant correlations were chosen to be depicted. A permissive threshold level of q ≤ 0.05 an r-value of ≥ 0.3 was chosen for the identification of significant correlations. Metabolites are color-coded and clustered according to the walktrap community algorithm. The significance of the communities with more than four nodes was tested by performing a Wilcoxon signed rank test. The test was performed by assessing the degree of node-connectivity of the isolated community as compared to the nodes of the community still embedded in the network of which all community specific edges have been subtracted. Positive correlations are denoted as blue edges, negative correlations are denoted as red edges. The sizes of the nodes represent the relative degree of connectivity. Computations of the correlations were conducted under the R environment. Cytoscape was used to generate graphical output of network. [file Image1.PDF]
